# The Plasma Bioavailability of Coenzyme Q_10_ Absorbed from the Gut and the Oral Mucosa

**DOI:** 10.3390/jfb9040073

**Published:** 2018-12-15

**Authors:** Luis Vitetta, Andrea Leong, Joyce Zhou, Serena Dal Forno, Sean Hall, David Rutolo

**Affiliations:** 1 The University of Sydney, Sydney Medical School, Faculty of Medicine and Health, Sydney, NSW 2006, Australia; 2 Medlab Clinical, Sydney, NSW 2015, Australia; andrea_leong@medlab.co (A.L.); joyce_zhou@medlab.co (J.Z.); serena_dalforno@medlab.co (S.D.F.) sean_hall@medlab.co (S.H.); david_rutolo@inmedtech.co (D.R.)

**Keywords:** Ubiquinone, Ubiquinol, Liposome, Coenzyme Q_10_ (CoQ_10_)

## Abstract

Coenzyme Q_10_ (CoQ_10_) has a central role in the generation of cellular bioenergy and its regulation. The hydrophobicity exhibited by the CoQ_10_ molecule leads to reports of poor absorption profiles, therefore, the optimization of formulations and modes of delivery is an ever-evolving therapeutic goal. The aim of this study was to investigate different CoQ_10_ formulations. The article summarizes the findings from an Australian comparative study involving adults administered CoQ_10_ through different oral delivery platforms. A total of 11 participants (six males and five females) voluntarily participated in a comparative clinical study of three different CoQ_10_ formulations across a six-week period, completing 198 person-hours of cumulative contribution equivalent to n = 33 participation. All of the eligible participants (n = 11) administered the three formulations blinded from who the commercial supplier of the formulation was and from what the chemical form of the CoQ_10_ was that was being administered. The dosing between the CoQ_10_ preparations were dispensed sequentially and were administered following three-week washouts. Three commercial preparations were tested, which included the following: formulations with capsules each containing ubiquinol and ubiquinone (150 mg/capsule), and a liposome ubiquinone formulation (40 mg/mL at 2 actuations of the pump). A significant inter-subject variation in the plasma level of CoQ_10_ at baseline that was observed to increase with an increase in age. This trend persisted in the post administration of the different formulations. Furthermore, it was observed that the intestinal absorption and bioavailability of CoQ_10_ varied significantly in the plasma between subjects, irrespective of whether the ubiquinol or ubiquinone forms were administered. The administration of CoQ_10_ as a liposome for preparation showed the poorest response in bioavailability. Although the ubiquinol capsule form of CoQ_10_ was observed to have increased in the plasma versus the ubiquinone capsules and the ubiquinol liposome at the two-hour interval, the inter-subject variation was such that the difference was not significant (*p* > 0.05). All of the CoQ_10_ formulations showed no further increases in their plasma levels over the remaining study period (i.e., four hours). This study further concluded that the intestinal absorption of CoQ_10_ is highly variable and is independent of the molecular form administered. Furthermore, it also concludes that liposomes are not an effective vehicle for the oral administration of CoQ_10_, and as such, did not improve the oral mucosal/sublingual absorption and bioavailability of the molecule. Of interest was the observation that with the increasing subject age, there was an observed increase in the baseline plasma CoQ_10_ levels in the participants prior to dosing. It was posited that the increase in the baseline plasma levels of CoQ_10_ with an increase in age could be due to the loss of skeletal muscle mass, a result that still needs to be verified.

## 1. Introduction

CoQ_10_ is a fat-soluble vitamin-like compound that is found throughout organ tissues, but particularly in the heart, skeletal muscle, liver, kidney, and brain [1]. CoQ_10_, as with all water-insoluble compounds (i.e., fat molecules), has been reported to be better absorbed in the intestines when administered after a meal containing fat [2]. The secretion of bile salt micelles by the liver into in the duodenum significantly improves the absorption of fats, leading to the formation of bile salt–mixed micelles complexes with the digestion products of dietary triglycerides (e.g., monoglycerides and fatty acids) [3]. The transport of lipophilic molecules through the aqueous environment of the intestines and across the unstirred water layer to the intestinal epithelia is a complex process that consists of intestinal digestion, uptake, intracellular metabolism, and the packaging of dietary lipids [4]. CoQ_10_ (ubidecarenone) is a biologically active compound that is analogous in its chemical structure to menaquinones (vitamin K_2_) [5]. 

CoQ_10_ is a member of the quinone family of compounds, characterized by a quinone ring attached to a repeating series of side-chain isoprene units (10 in CoQ_10_). CoQ_10_ is an insoluble compound that has a central role in the bioenergy metabolism, and participates in the generation of proton motive force (i.e., energy) in closed membrane systems [6]. As such, CoQ_10_ is often classified as an antioxidant molecule [1]. Antioxidants have been defined as molecules or compounds that delay, prevent, or remove oxidative damage to a target molecule [7]. Notwithstanding, there are no clinical studies that unambiguously demonstrate an over production of systemic free radicals that lead to chronic disease development. Furthermore, there are no human studies that explicitly show that small molecule antioxidants can abrogate free radicals in the systemic circulation under normal physiological conditions. Therefore, CoQ_10_ is a molecule that our group believes has largely been mislabeled as an anti-oxidant, as it has no clearly defined antioxidant effects in vivo, other than to transfer electrons across the electron transport chain within mitochondria and cellular/plasma membranes [6,8].

Notwithstanding, CoQ_10_ has been reported to provide beneficial effects in a number of chronic diseases [9]. The community has hence shown significant interest in administering formulations containing CoQ_10_, and companies marketing CoQ_10_ products have also often made exorbitant efficacy claims surrounding its antioxidant effects and absorption characteristics from the gut. Moreover, as knowledge about the biochemistry of statin medications became widely available, relative to the its beneficial effects on blood lipid metabolism, adverse effects on skeletal muscle have been recently widely reported [10]. This, further fuels the belief that CoQ_10_ may have myalgia/myopathy curative properties [11]. Analogous results have led CoQ_10_ marketers to further justify and help progress the clinical posit for the concomitant administration of CoQ_10_ with a statin, in order to prevent adverse muscle symptoms [12]. Yet the clinical evidence does not support the industry view regarding the beneficial effects of CoQ_10_ on skeletal muscle [13]. Furthermore, studies have queried the absorption of CoQ_10_ in the intestines by investigating novel routes of administration [14].

The aim of this limited clinical study was to compare the absorption characteristics over a six-hour period of three different CoQ_10_ formulations administered via the oral-intestinal tract (e.g., CoQ_10_ capsules) and the oral buccal mucosa [15,16,17] (e.g., liposome CoQ_10_), and did not aim to reproduce a pharmacokinetic study. 

## 2. Methods

### 2.1. Subjects/Blood Samples Collected/Formulations Administered

Samples of peripheral venous blood were collected via venipuncture by a registered nurse trained in phlebotomy, using a 19-gauge, 1-inch multi-use needle into a 5 mL green-topped (heparin) Vacutainer® tube. A total of 213 blood samples were collected from a group of healthy volunteers (n = 6 males and n = 5 females). None of the participants that were invited to participate in the study had a serious or chronic disease diagnosis on induction, nor were they administered any medications at the time of the study; if they were administered any form of supplements at the time of enrolment, they were instructed to cease one week prior to participation and during blood sampling. The participant demographics are presented in Table 1. Written informed consent was obtained from each participant before starting the protocol. All of the participants adhered to an overnight fast and consented to be repeat participants in the clinical study.

Three formulations were investigated across a six-week period, including the following: (i) a 150 mg ubiquinol (the fully reduced form) capsule, (ii) a 150 mg ubiquinone (fully oxidized) capsule, and (iii) a liposome formulation (ubiquinol 40 mg/two sprays). The formulations chosen comprise those that were readily available and were sold by well-known nutraceutical suppliers in Australia, and were commonly purchased by the community.

The participants all administered the three formulations with three-week washout periods between formulations, prior to recommencing the dosing schedule with the subsequent preparations. The study was conducted in the morning following an overnight fast, with blood sample collections beginning on the morning of the test at 8:00 am, and continuing for six hours. A six-hour study time was chosen as suitable for investigating the absorption characteristics of the different CoQ_10_ formulations. Furthermore, an interrogation of the published literature showed that the pharmacokinetic studies with CoQ_10_ showed that Tmax was approximately 6 h [13].

Following the baseline blood draw and CoQ_10_ dose administration, the participants were provided and encouraged to consume the same simple continental breakfast. The subjects were queried about their recent physical activity regimens, and all reported similar levels of activity within the previous week prior to commencing participation in the study.

The clinical study was not randomized and the enrolled subjects that were aware that they were dosing with CoQ_10_. However, all of the participants were blinded as to the identity of the form of the CoQ_10_, namely, either ubiquinol or ubiquinone, being tested. Moreover, the participants were also blinded as to who the commercial supplier of the CoQ_10_ formulation was.

The National Institute of Integrative Medicine Human Research Ethics Committee (HREC) [0035_2016] approved the study. Following HREC approval, the clinical trial received CTN authorization from the Therapeutic Goods Administration (CT-2017-CTN-03598-1 v1) in order to proceed. The clinical trial was registered with the Australian and New Zealand Clinical Trial Registry (ACTRN12616001527459).

The blood samples were centrifuged at 3000 *g* for 20 min at 4 °C. The plasma was separated from the blood cells and was immediately stored in 1.5 mL micro-centrifuge tubes at −80 °C. 

### 2.2. Plasma CoQ_10_ Assay

The plasma CoQ_10_ was quantified using an enzyme-linked immunosorbent assay (ELISA) technique with commercially available kits from Biomatik, according to the manufacturer’s instructions. The detection concentration range of the CoQ_10_ in plasma with the Biomatik kits was 3.12 ng/mL–50 ng/mL. In brief, the microtiter plate provided in the kit was pre-coated with an antibody specific to CoQ_10_. Standards or samples were added to the appropriate microtiter plate wells with horseradish peroxidase conjugated CoQ_10_. The competitive inhibition reaction was launched between the horseradish peroxidase-conjugated CoQ_10_ and the CoQ_10_ in the samples. A substrate solution was added to the wells and the color developed in contrast to the amount of CoQ_10_ in the sample. The color development was stopped and the intensity of the color was measured. The plasma samples were diluted, using the supplied assay diluent, in order to bring the CoQ_10_ concentrations into the range measurable by the kit (a dilution factor of 225 was found by a pilot experiment to be appropriate for the samples). The CoQ_10_ concentrations of the samples were diluted as determined by the manufacturer’s instructions, with reference to a standard curve that was included on each ELISA plate. The original concentration of CoQ_10_ in the plasma samples was calculated by multiplying these results by 225. 

### 2.3. Statistical Analysis

The demographic variables were represented as means of the standard deviation (SD). The CoQ_10_ values determined in the plasma comprised a continuous variable, and, given that the data were skewed and not normally distributed, the results were presented as medians (interquartile ranges). The results were graphically represented as box plots in order to demonstrate the changes in the plasma levels of CoQ_10_, from baseline through to 30, 60, 120, 240, and 360 min post administration of the controlled investigational dose of a CoQ_10_ formulation. Nonparametric tests (Kruskal-Wallis) were used to assess whether there were significant association effects present from baseline to six hours for the three different CoQ_10_ formulations administered. 

The mean (SD) plasma levels of CoQ_10_ were also calculated. All of the data analyses and graphical representations were conducted with Stata Corporation Software version 15.1. 

## 3. Results

Males (n = 6) and females (n = 5) comprising a ratio of approximately 1 to 1 volunteered to participate in a comparative absorption study of three CoQ_10_ formulations. 

**Figure 1:**
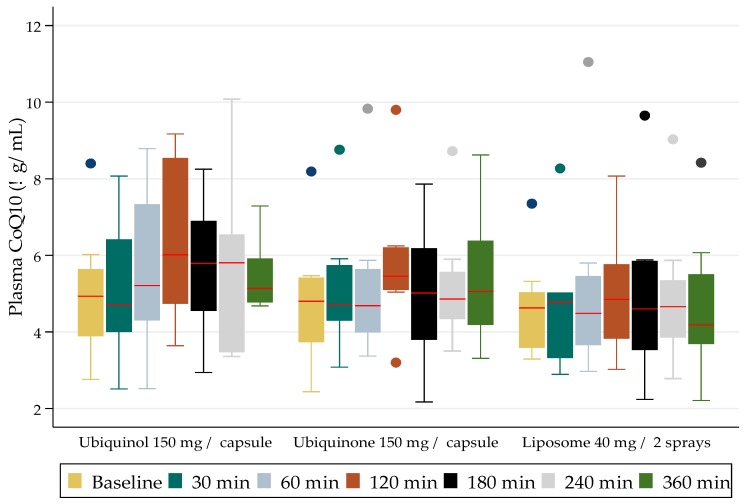
Median (interquartile range) CoQ_10_ plasma levels and dispositions from baseline to 6 h for the three formulations tested.

The participants provided a total of 231 plasma samples for the CoQ_10_ assays over the course of the clinical study, namely six weeks. The comparisons of the mean (SD) levels achieved (red line) over a six-hour study period are presented in Figure 1. 

**Figure 2:**
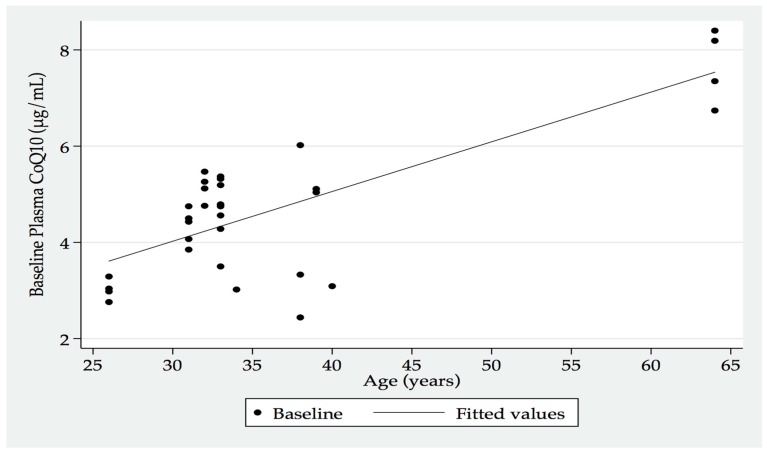
Baseline plasma CoQ_10_ by age of participants.

An interesting corollary from this study, albeit the small participant numbers, was the increasing baseline plasma CoQ_10_ levels that were recorded with the increasing age of the participants (Figure 2). This factor was posited to perhaps indicate an increased release of cellular CoQ_10_ into the systemic circulation as a consequence of tissue loss with ageing (e.g., skeletal muscle mass loss).

The dosing results (presented graphically in Figure 1) demonstrate the compared blood level concentrations of CoQ_10_ achieved over the six-hour study time with each formulation. The CoQ_10_ as ubiquinol (reduced form) delivered as a 150 mg capsule, showed the highest increase at two hours. The difference, however, was not statistically significant when compared to the levels achieved at two-hours with the other formulations. The formulation that performed the poorest was the CoQ_10_ oral delivered liposome. No adverse events were observed after the administration of each formulation.

The mean (SD) levels for the 150 mg ubiquinol formulation ranged from 5 (0.6) μg/mL at baseline to 5.5 (0.3) μg/mL at six-hours, with a peak value at two-hours of 6.4 (0.8) μg/mL. The mean (SD) levels for the 150 mg ubiquinone formulation ranged from 4.8 (0.6) μg/mL at baseline to 5.4 (0.7) μg/mL at six-hours, with a peak value at two-hours of 5.8 (0.7) μg/mL. The mean (SD) levels for the 40 mg liposome formulation ranged from 4.8 (0.5) μg/mL at baseline to 5.0 (0.7) μg/mL at six-hours, with a peak value at two-hours of 5.3 (0.6) μg/mL. There were no statistically significant differences between the formulations.

## 4. Discussion

The data presented from this study suggests that the CoQ_10_ formulations based on oral/digestive tract and liposome delivery systems that are currently available on the Australian market place did not improve the enteral or oral mucosa absorption and bioavailability in a healthy cohort of males and females. These results further confirm that compounds such as CoQ_10_ that exhibit poor oral bioavailability are due to their rate-limiting solubility.

The formulations for the delivery of compounds, namely nutritional compounds (e.g., ascorbate, magnesium) or active pharmaceutical ingredients, have been marketed in various platforms, from capsules/tablets to lozenges to powders and emulsions/liquids. The intestinal absorption can be dependent on the intrinsic and extrinsic environmental variables [18]. Hence, it is very much recognized that a healthy-functioning intestinal system will ensure the adequate absorption in the intestinal tract [18]. Nevertheless, although this study showed that with highly insoluble active ingredients, such as CoQ_10_, there was a poor bioavailability profile that was observed. Nano-pharmaceuticals deliver compounds in platforms on the basis of small particle sizes, and these delivery systems may have therapeutic applications in multiple areas [19]. 

A lipid-based formulation of CoQ_10_ has been previously shown to be more bioavailable in plasma than that of other orally administered formulations [14]. The reported mean (SD) total plasma CoQ_10_ has been reported to be 0.9 (0.3) μg/L [20]. Such reports have given way to the development of nanotechnology-based formulations, such as liposomes. However, liposomes may not be a suitable delivery platform, given the limited safety data [21] and toxicity associated drawbacks [22,23] that have been reported. The formulation optimization of CoQ_10_ has historically been driven by gut absorption issues [24], and appears to be in deficit.

We have previously reported on the absorption and blood levels achieved with orally administered vitamin B_12_ formulations [25]. In that study, we investigated a nanotechnology-based micelle formulation, developed to capture the idea of exploiting the delivery of organic molecules at the nanometre scale via the oro-buccal mucosa [25]. Oro-buccal delivered formulations, such as that achieved with vitamin B_12_, demonstrated a more enhanced bioavailability than capsules, tablets, or a liposome formulation. As such, our research group remains committed to further investigating an oro-buccal delivered nanoparticle formulation for the enhanced delivery of CoQ_10_ to intracellular and mitochondrial targets. *In vitro* and *in vivo* studies have recently demonstrated that electro-spraying microparticles may comprise a possible future technology for improving the bioavailability of compounds such as CoQ_10_ [26].

The loss of muscle mass with increasing age is an inevitable step in the ageing process. A loss of muscle mass is common in the elderly, especially as it is associated with increased frailty, which leads to increased dependence and, eventually, to mortality [27]. Further changes are readily noticeable, for example, in voice changes in the elderly, predominantly due to larynx muscle atrophy [28]. CoQ_10_ has a fundamental participatory role in cellular bioenergetics [13], and as a co-factor in the mitochondrial biogenesis processes of energy production and cellular redox potential equilibrium [6]. It is hence possible to cautiously speculate that the administration of CoQ_10_ (as the reduced form, namely ubiquinol/hydroquinone) earlier in life may have beneficial effects relative to slowing the progression of muscle mass loss with increasing age. The administration of CoQ_10_ in its reduced form is consistent with reports that show that most of the CoQ_10_ that is found in tissues is in the reduced form [29]. 

## 5. Conclusions

Investigating the pharmacokinetics of the CoQ_10_ formulations was beyond the scope of the present study. CoQ_10_ pharmacokinetic studies have reported a Tmax of approximately six hours [13], hence the intention of this study was solely to observe and report the early disposition of the molecule. Three different common commercially available preparations were investigated, given the high interest from the lay and clinical community to the administer CoQ_10_. 

Studies that have investigated the administration of CoQ_10_ to subjects undergoing elective hip replacement surgery demonstrated that CoQ_10_ treatment influenced the fiber type composition towards the fiber type profile generally found in younger individuals in the vastus lateralis muscle samples [30]. Such studies provide renewed research incentives to further investigate non-traditional routes of active molecule delivery to tissues such as skeletal muscles. Nanotechnology-based delivery systems present platforms that could allow for the safe and efficacious delivery of poorly intestinally absorbed molecules, such as CoQ_10_. We have previously documented the safety, tolerability, and efficacious delivery of a vitamin complex in a micelle nanoparticle delivery system [25], and as such, provide further knowledge to progress the basic research efforts to a nanoparticle CoQ_10_ formulation. 

## Figures and Tables

**Table 1:** Participant demographics at baseline. BMI—body mass index; BP—blood pressure; SD—standard deviation.

Demographic Variables	Males (6) means (SD)	Females (5) means (SD)
Age (years)	37.7 (13.6)	35 (3.9)
BMI (Kg/m^2^)	27.8 (4.3)	22.9 (2.1)
Systolic BP (mmHg)	138.2 (14.6)	117.2 (9.8)
Diastolic BP (mmHg)	88 (9.9)	76.8 (3.9)
Allergies		
— Yes	1	0
— No	5	5
